# Association of ADAMTS13 polymorphism with cerebral malaria

**DOI:** 10.1186/1475-2875-10-366

**Published:** 2011-12-14

**Authors:** Sirima Kraisin, Izumi Naka, Jintana Patarapotikul, Duangdao Nantakomol, Pornlada Nuchnoi, Hathairad Hananantachai, Naoyuki Tsuchiya, Jun Ohashi

**Affiliations:** 1 Doctoral Program in Biomedical Sciences, Graduate School of Comprehensive Human Sciences, University of Tsukuba, Ibaraki, Japan; 2 Master of Science Program in Clinical Hematology Sciences, Department of Clinical Microscopy, Faculty of Allied Health Sciences, Chulalongkorn University, Bangkok, Thailand; 3 Department of Microimmunology and Clinical Tropical Medicine, Faculty of Tropical Medicine, Mahidol University, Bangkok, Thailand; 4 Department of Clinical Microscopy, Faculty of Medical Technology, Mahidol University, Bangkok, Thailand

## Abstract

**Background:**

Cerebral malaria is one of the most severe manifestations of *Plasmodium falciparum *malaria. The sequestration of parasitized red blood cells (PRBCs) to brain microvascular endothelium has been shown to contribute to the pathophysiology of cerebral malaria. Recent studies reported increased levels of von Willebrand factor (VWF) and reduced activity of VWF-cleaving protease, ADAMTS13 (a disintegrin and metalloproteinase with a thrombospondin type 1 motif, member 13), in patients with cerebral malaria.

**Methods:**

Association of six single nucleotide polymorphisms (SNPs) of the *ADAMTS13 *gene with cerebral malaria was examined in 708 Thai patients with *P. falciparum *malaria.

**Results:**

Among six SNPs, the derived allele of a SNP located in intron 28, rs4962153-A, was significantly associated with protection against cerebral malaria when 115 cerebral malaria patients were compared with 367 mild malaria patients (Fisher's exact *P*-value = 0.0057; OR = 0.27; 95% CI = 0.096-0.76). Significant association was also detected between 115 cerebral malaria and 593 non-cerebral malaria (226 non-cerebral severe malaria and 367 mild malaria) patients (Fisher's exact *P*-value = 0.012; OR = 0.30; 95% CI = 0.11-0.83).

**Conclusions:**

Excessive adhesion of PRBCs to the platelet-decorated ultra-large VWF (ULVWF) appears to enhance the sequestration of PRBCs to cerebral microvascular endothelium. The genetic association observed in the present study implies that the regulation of platelet-decorated ULVWF strings by ADAMTS13 may play a role in the development of cerebral malaria.

## Background

Malaria is a major cause of morbidity and mortality in tropical countries. Approximately 250 million clinical cases of malaria occur, resulting in almost 1 million deaths each year. Cerebral malaria, one of the most serious complications of *Plasmodium falciparum *malaria infection, is an important cause of death. The excessive sequestration of parasitized red blood cells (PRBCs) to brain microvascular endothelium has been suggested to specifically contribute to the pathophysiology of cerebral malaria [1], yet the pathogenesis of cerebral malaria remains poorly understood.

It has been demonstrated that levels of von Willebrand factor (VWF) are significantly increased in malaria patients compared with non-malarial patients with fever or control subjects [2]. Further studies confirmed release of VWF after endothelial cell activation in patients with *falciparum *malaria [3-5] and detected adhesion of PRBCs to platelet-decorated ultra-large VWF (ULVWF) strings [3]. The platelet-decorated strings are cleaved and regulated by plasma protease ADAMTS13 (a disintegrin and metalloproteinase with a thrombospondin type 1 motif, member 13) [6,7]. Together with the presence of abnormal circulating ULVWF multimers, a significant reduction in plasma ADAMTS13 function was also observed in cerebral or severe malaria patients compared to healthy controls [5]. These observations suggest that decreased ADAMTS13 activity plays a crucial role in the development of cerebral malaria.

The human *ADAMTS13 *gene (OMIM *604134), spanning approximately 45 kb, is located on chromosome 9q34 [6,7]. To date, a number of *ADAMTS13 *mutations causing congenital thrombotic thrombocytopaenic purpura (TTP), a rare life-threatening disease characterized by thrombocytopenia, microangiopathic haemolytic anaemia, renal failure, and neurological deficits including coma, have been identified [6,8]. These symptoms are also key features of severe *falciparum *malaria. Although *ADAMTS13 *is a candidate gene for cerebral malaria, no genetic association study of *ADAMTS13 *in malaria patients has been conducted. This study reports a significant association of common *ADAMTS13 *polymorphism with protection against cerebral malaria in Thai patients with *falciparum *malaria.

## Methods

### Subjects

A total of 708 Thai patients infected with *P. falciparum *living in north-west of Thailand, near the border with Myanmar, were recruited in this study. Malarial infection by *P. falciparum *was confirmed by a positive blood smear for the presence of asexual form of *P. falciparum*. Clinical manifestations of malaria were classified according to the definitions and associated criteria by the World Health Organization. The mild malaria group consisted of 367 patients with mild symptoms characterized by fever without other causes of infection and by lack of the manifestations of severe malaria as described below. The non-cerebral severe malaria group comprised 226 patients with severe malaria which was defined as one with one of the following signs of severity or evidence of vital organ dysfunction: high parasitaemia (> 100,000 parasites/μL), hypoglycaemia (glucose level < 22 nmol/L), severe anaemia (haematocrit < 20% or haemoglobin level < 7 g/dL), a serum creatinine level > 3.0 mg/dL. The cerebral malaria group consisted of 115 patients characterized by an unrousable coma and exclusion of other causes of coma. The patients were ≥13 years old, and the average ages of the patients with mild malaria, non-cerebral severe malaria and cerebral malaria were 25.2, 20.5 and 28.6 years, respectively. All patients underwent treatment at the Hospital for Tropical Disease, Faculty of Tropical Medicine, Mahidol University (Bangkok, Thailand). This study was approved by the institutional review board of Faculty of Tropical Medicine, Mahidol University, and the Research Ethics Committee of the Graduate School of Comprehensive Human Sciences, University of Tsukuba. Written informed consent was obtained from every patient.

### DNA extraction

Genomic DNA was extracted from peripheral blood leukocytes using a QIAamp blood kit according to the manufacturer's instruction (QIAGEN, Hilden, Germany).

### SNP selection

Based on the SNP genotype data of the Asian HapMap samples, 45 Japanese in Tokyo, Japan (JPT) and 45 Han Chinese in Beijing, China (CHB) [9,10], five SNPs with minor allele frequency of more than 0.05 were selected as tag SNPs from 15 SNPs of the *ADAMTS13 *gene by using the Tagger algorithm implemented in the Haploview software version 4.2 with the default settings (i.e., pairwise tagging only with linkage disequilibrium (LD) parameter, *r*^2^, threshold of ≥ 0.8). Accordingly, five SNPs were selected as tag SNPs (Table 1). In addition, a synonymous SNP, rs1055432, was also selected since the FastSNP Search, a web-based tool providing the potential functional effect of SNP, identified it as a SNP with low-medium risk.

**Table 1:** SNPs analysed in this study

rs#^a^	HGVS name^a, b^	Chromosome coordinate^a^	Region^a^	Ancestral allele/derived allele^a ^(Allele frequency^c^)
rs2301611	g.8488T >C	136290607	intron 3	T (0.854)/C (0.146)

rs3118667	g.8944C >T	136291063	exon 5	T (0.803)/C (0.197)

rs739469	g.16610G >C	136298729	intron 10	C (0.213)/G (0.787)

rs652600	g.28898G >A	136311017	intron 20	T (0.247)/C (0.753)

rs4962153	g.41635A >G	136323754	intron 28	G (0.921)/A (0.079)

rs1055432	g.42120C >A	136324239	exon 29	C (0.750^d^)/A (0.250)

Note; *HGVS *(Human Genome Variation Society) name

^a ^The SNP reference number, HGVS name, chromosome coordinate, region, and ancestral state were retrieved from the National Center for Biotechnology Information (NCBI) SNP database build 132, September 2010.

^b ^The position of SNP was determined according to the HGVS nomenclature based on the NCBI reference sequence NG_011934.1.

^c ^The allele frequency in the Asian HapMap populations (JPT+CHB).

^d ^The Asian HapMap samples were genotyped in this study.

### Genotyping

The TaqMan SNP Genotyping Assay was used to genotype six *ADAMTS13 *SNPs (Table 1) for 708 Thai malaria patients using 7300 Real-Time PCR System (Applied Biosystems). In this study, rs1055432 was genotyped for the Asian HapMap samples (HapMap-JPT + CHB) to investigate the LD structure of *ADAMTS13*. A nonsynonymous SNP (rs11575933, P475S) located in exon12 of *ADAMTS13 *was analysed in 128 Thai malaria patients (64 from mild malaria group and 64 from cerebral malaria group) by using the polymerase chain reaction (PCR)-direct sequencing. The PCR was performed using the following primers: 5'-ACCCAGCTGGAGTTCATGTC-3' and 5'-CCTATGACTCTGCCCTGTCC-3', and the PCR products were subjected to direct sequencing with ABI prism 3100 Genetic Analyzer (Applied Biosystems).

### Statistical analyses

Deviation from Hardy-Weinberg equilibrium was tested in each malaria group by chi-square test. The allele frequency was compared between two malaria groups by Fisher's exact test. The odds ratio (OR) and 95% confidence interval (CI) were calculated by Woolf's method. In this study the direction of association was determined based on the derived allele regardless of the allele frequency. *P*-value ≤ 0.05 was considered statistically significant. To address the problem of multiple testings, permutation *P*-values were calculated for single SNPs from 100,000 permutations using the Haploview software, version 4.2. Haplotype frequency and pairwise LD parameter, *r*^2^, were estimated using the Haploview software. The haplotype association test based on the permutation procedure was conducted using the Haploview software. In the haplotype association test, to find a haplotype showing stronger association with cerebral malaria than SNP, permutation *P*-values were calculated for single SNPs and haplotypes from 100,000 permutations.

The association of rs4962153 with platelet count obtained from 449 malaria patients before treatment (on Day 0) was assessed by analysis of covariance (ANCOVA) adjusted for age (years), gender (female or male), and symptom (mild, non-cerebral severe, or cerebral), where dummy variables were used for symptom.

## Results

### Association test

**Figure 1:**
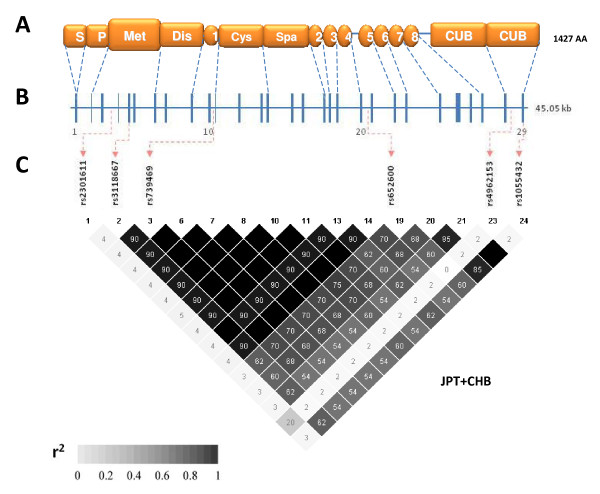
**Structure of the *ADAMTS13 *gene and LD plot**. **A**, ADAMTS13 protein (1427 amino acids) consisting of a signal peptide (*S*), a propeptide (*P*), a metalloprotease domain (*Met*), a disintegrin-like domain (*Dis*), a thrombospondin type-1 (TSP-1) repeat (*1*), a cysteine-rich domain (*Cys*), a spacer domain (*Spa*), seven additional TSP-1 repeats (*2-8*), and two complement C1r/C1s, Uegf, Bmp1 (*CUB*) domains. **B**, *ADAMTS13 *gene comprising 29 exons on the human chromosome 9q34. **C**, LD plot among 15 SNPs with minor allele frequency of more than 0.05 in the Asian HapMap populations (JPT + CHB). Five tagSNPs and rs1055432 analysed in Thai malaria patients are indicated by arrows. A pairwise *r*^2 ^value is shown in each square. Darker shading indicates higher *r*^2 ^value and black shading indicates *r*^2 ^of 1.

Six SNPs, two in exon and four in intron, of the *ADAMTS13 *gene were analysed in this study (Table 1 and Figure 1a). All the SNPs did not deviate from Hardy-Weinberg equilibrium in each malaria group (Table 2). Association analyses revealed that rs4962153-A (derived allele of rs4962153) was strongly associated with protection against cerebral malaria, when mild and cerebral malaria groups were compared (Fisher's exact *P*-value = 0.0057; OR = 0.27; 95% CI = 0.096-0.76; Table 2). The *P*-value was still significant even in permutation test for six SNPs in the comparison between mild and cerebral malaria groups (Permutation *P*-value = 0.041). There was no significant difference in allele frequency of rs4962153-A between non-cerebral severe and cerebral malaria groups (*P*-value = 0.082), although a slight tendency towards protection against cerebral malaria was observed in this comparison as well. When mild and severe malaria groups were combined as a non-cerebral malaria group, the frequency of rs4962153-A was significantly higher in non-cerebral malaria than in cerebral malaria (Fisher's exact *P*-value = 0.012; OR = 0.30; 95% CI = 0.11-0.83), while the permutation *P*-value was not significant.

**Table 2:** Allele frequencies of *ADAMTS13 *SNPs in Thai malaria patients

SNP	Allele frequency			Association *P*-value		
	
	M (2n = 734)	S (2n = 452)	C (2n = 230)	M vs C	S vs C	M + S vs C^a^
rs2301611;						
T	691 (0.94)	430 (0.95)	223 (0.97)			
C	43 (0.06)	22 (0.05)	7 (0.03)	0.12	0.32	0.14
HWE *P*-value	0.23	0.44	0.74			

rs3118667;						
T	523 (0.71)	297 (0.66)	163 (0.71)			
C	211 (0.29)	155 (0.34)	67(0.29)	0.93	0.19	0.64
HWE *P*-value	0.74	0.31	0.73			

rs739469;						
C	525 (0.72)	298 (0.66)	160 (0.70)			
G	209 (0.28)	154 (0.34)	70 (0.30)	0.56	0.34	1.0
HWE *P*-value	0.41	0.26	0.88			

rs652600;						
T	470 (0.64)	273 (0.60)	152 (0.66)			
C	264 (0.36)	179 (0.40)	78 (0.34)	0.58	0.16	0.33
HWE *P*-value	0.14	0.32	0.25			

rs4962153;						
G	689 (0.94)	431 (0.95)	226 (0.98)			
A	45 (0.06)	21 (0.05)	4 (0.02)	0.0057^b^	0.082	0.012^c^
HWE *P*-value	0.14	0.44	0.84			

rs1055432;						
C	516 (0.70)	293 (0.65)	160 (0.70)			
A	218 (0.30)	159 (0.35)	70 (0.30)	0.87	0.23	0.76
HWE *P*-value	0.16	0.76	0.55			

Note; HWE, Hardy-Weinberg equilibrium; M, mild malaria; S, non-cerebral severe malaria; C, cerebral malaria.

^a ^Since the frequency of rs4962153-A was different between mild and cerebral malaria groups, the frequency was further compared between non-cerebral (mild and non-cerebral severe malaria groups) and cerebral malaria groups.

^b ^OR = 0.27; 95% CI = 0.096-0.76; Permutation *P*-value = 0.041.

^c ^OR = 0.30; 95% CI = 0.11-0.83; Permutation *P*-value = 0.074.

The SNP rs11575933 (P475S) has been reported to influence the proteolytic activity of ADAMTS13 [11-13]. In 128 Thai malaria patients, rs11575933-T (475S) allele was not observed. Thus, the remaining samples were not analysed, and rs11575933 was not considered in the association analysis.

### LD structure and haplotype frequency

**Figure 2:**
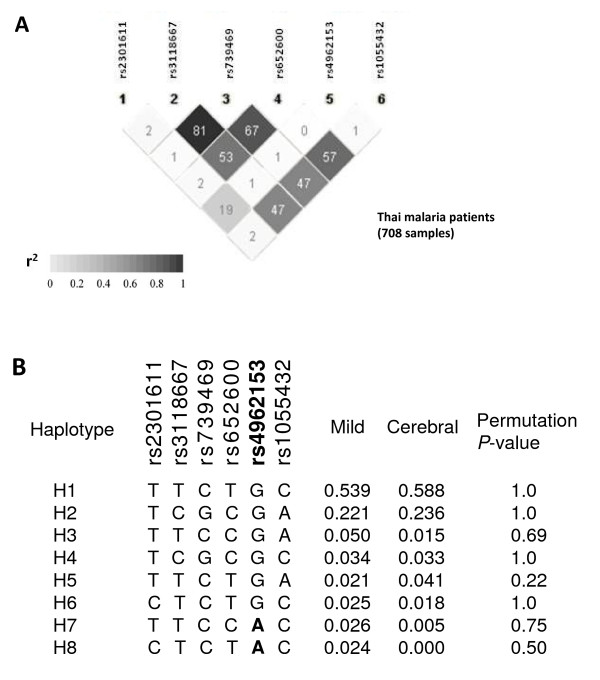
**LD plot and haplotype frequency in Thai malaria patients**. **A**, LD plot among six SNPs analysed in 708 Thai malaria patients. A pairwise *r*^2 ^value is shown in each square. Darker shading indicates higher *r*^2 ^value. **B**, Haplotype frequencies in mild and cerebral malaria patients. Only haplotypes with frequency of more than 0.02 either in mild or in cerebral malaria patients are shown. Permutation *P*-values were calculated for single SNPs and haplotypes from 100,000 permutations.

The pairwise *r*^2 ^values among six *ADAMTS13 *SNPs were not high in this study (Figure 2a). This is mainly because they were selected as tag SNPs based on the Asian HapMap populations (JPT + CHB). To evaluate whether any *ADAMTS13 *haplotype consisting of six *ADAMTS13 *SNPs shows stronger association with cerebral malaria than rs4962153, permutation *P*-values for single SNPs and haplotypes were further calculated in the comparison between mild and cerebral malaria groups. In this setting, the permutation *P*-value of rs4962153 was 0.080, and no haplotype showed permutation *P*-value lower than rs4962153 (Figure 2b).

Haplotype analysis indicated that rs4962153-A was on H7 and H8 haplotypes (Figure 2b). The estimated frequencies of these haplotypes in mild malaria patients were higher than those in cerebral malaria patients, suggesting that the significant association of rs4962153-A with protection against cerebral malaria was not caused by either one of the haplotypes bearing rs4962153-A.

### Platelet counts and rs4962153

**Figure 3:**
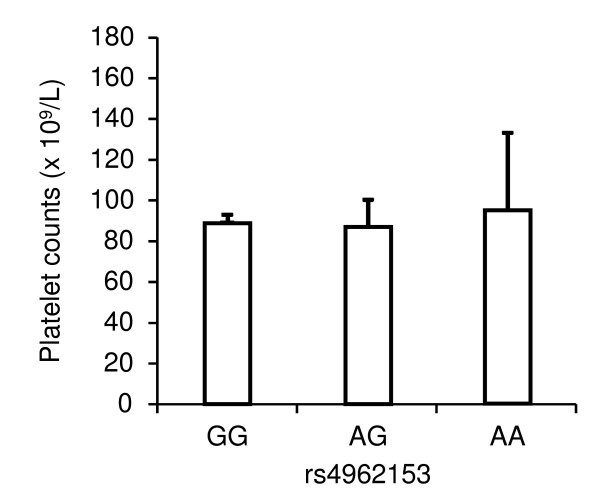
**The means and standard errors of platelet counts broken down by rs4962153 genotypes**. Comparison among AA, AG, and GG genotypes at rs4962153 was made using ANCOVA by adjusting for age (years), gender (female or male), and symptom (mild, non-cerebral severe, or cerebral). No significant difference was detected (*P*-value = 0.98).

Since platelet-decorated ULVWF strings are cleaved and regulated by ADAMTS13 [6,7], rs4962153 may influence platelet counts in malaria patients. To evaluate this, ANCOVA was conducted. The values adjusted for age, gender, and symptom are shown in Figure 3. The result indicated that platelet counts were not significantly different among AA, AG, and GG genotypes at rs4962153 (*P*-value = 0.98).

## Discussion

The reduced activity of ADAMTS13 in malaria patients may allow overproduction of ULVWF multimers. The following adhesion of PBRCs to platelet-decorated ULVWF strings would result in excessive sequestration of PBRCs to brain microvascular endothelium. This has been proposed as one of important mechanisms of adhesion of PBRCs to endothelium in cerebral malaria [14]. The present study revealed that rs4962153-A of *ADAMTS13 *was significantly associated with protection against cerebral malaria in 708 Thai malaria patients. The genetic association observed in the present study supports the above-mentioned role of ADAMTS13 in the development of cerebral malaria, although the functional significance of rs4962153-A remains to be studied. A better understanding of the regulation of platelet-decorated ULVWF strings by ADAMTS13 would provide new insights into the development of the treatment of cerebral malaria. For instance, to eliminate ULVWF and replace ADAMTS13, plasma exchange might potentially be considered a new treatment.

A previous study has shown that not only plasma ADAMTS13 activity but also plasma ADAMTS13 concentration were reduced in cerebral or severe malaria patients compared to healthy controls [5]. Since the frequency of rs4962153-A was higher in mild malaria patients than in cerebral malaria patients, rs4962153-A may enhance the plasma level of ADAMTS13, although platelet counts were not affected by rs4962153-A (Figure 3). A recent study reported that rs4962153-A was significantly associated with susceptibility to ischemic stroke in Swedish [15]. Considering that plasma levels of ADAMTS13 have been reported to be decreased in patients with myocardial infarction [16,17], rs4962153-A may reduce the plasma ADAMTS13 concentration in patients with ischemic stroke. Since it seems unlikely that the transcription level of *ADAMTS13 *is capable of being increased and decreased by rs4962153-A depending on the situation, the detected association of rs4962153-A may be a false positive in either study. To examine the present results, the effect of rs4962153 on the transcription and/or plasma levels of *ADAMTS13 *in malaria patients and healthy controls should be investigated in the future.

Since variation screening of the *ADAMTS13 *gene for Thai malaria patients was not conducted, the possibility that the association between rs4962153 and cerebral malaria detected in the present study has been caused by LD from the other polymorphisms cannot be excluded. The analysis of LD based on the HapMap database revealed that, in the Asian HapMap populations (JPT + CHB), rs4962153 was in strong LD with rs739468, rs3124765, and rs3094379 with *r*^2 ^of 1, 1, and 0.86, respectively. These three SNPs are on the chromosome 9 open reading frame 7 (*C9orf7*) gene, located adjacent to *ADAMTS13*. Flower protein, the ortholog of C9orf7 in *Drosophila, *has been suggested to function as a Ca^2+ ^channel to control Ca^2+ ^influx in insect salivary gland [18]. The function of human *C9orf7 *gene remains to be studied. However, it appears unlikely that polymorphism of *C9orf7 *is primarily associated with cerebral malaria, since Ca^2+ ^channel is not currently implicated in the pathogenesis of cerebral malaria. Since this is the first study reporting the significant association of *ADAMTS13 *polymorphism with cerebral malaria, further independent studies are required to examine whether the present finding can be replicated.

## Conclusions

PRBCs adhere to the platelet-decorated ULVWF, leading to enhancement of the sequestration of PRBCs to cerebral microvascular endothelium. The platelet-decorated ULVWF strings are cleaved and regulated by ADAMTS13. It has been reported that plasma ADAMTS13 function is significantly reduced in cerebral malaria patients compared to healthy controls. In this study, a SNP of *ADAMTS13*, rs4962153, was found to be significantly associated with cerebral malaria in 708 Thai patients with *P. falciparum *malaria. The present findings support the hypothesis that the regulation of platelet-decorated ULVWF strings by ADAMTS13 may play a role in the development of cerebral malaria.

## Competing interests

The authors declare that they have no competing interests.

## Authors' contributions

SK participated in the design of this study, carried out the genotyping, conducted statistical analyses, and wrote the manuscript. IN helped to genotype the samples. IN, JP, DN, PN and HH collected blood samples and extracted DNA from the samples. JP participated in the design of the study and coordination. NT was involved in the interpretation of the data and preparation of the manuscript. JO conceived of the study, participated in its design, helped to perform statistical analyses, and helped to write the manuscript. All authors read and approved the final manuscript.
